# Molecular Characteristics of Pancreatic Ductal Adenocarcinoma

**DOI:** 10.4061/2011/620601

**Published:** 2011-03-27

**Authors:** Niki A. Ottenhof, Roeland F. de Wilde, Anirban Maitra, Ralph H. Hruban, G. Johan A. Offerhaus

**Affiliations:** ^1^Department of Pathology, University Medical Center Utrecht, 3584 CX Utrecht, The Netherlands; ^2^Department of Pathology, The Sol Goldman Pancreatic Cancer Research Center, The Johns Hopkins Medical Institutions, Baltimore, MD 21287, USA

## Abstract

Pancreatic cancer is an almost universally lethal disease and despite extensive research over the last decades, this has not changed significantly. Nevertheless, much progress has been made in understanding the pathogenesis of pancreatic ductal adenocarcinoma (PDAC) suggesting that different therapeutic strategies based on these new insights are forthcoming. Increasing focus exists on designing the so-called targeted treatment strategies in which the genetic characteristics of a tumor guide therapy. In the past, the focus of research was on identifying the most frequently affected genes in PDAC, but with the complete sequencing of the pancreatic cancer genome the focus has shifted to defining the biological function that the altered genes play. In this paper we aimed to put the genetic alterations present in pancreatic cancer in the context of their role in signaling pathways. In addition, this paper provides an update of the recent advances made in the development of the targeted treatment approach in PDAC.

## 1. Pancreatic Ductal Adenocarcinoma

Annually, approximately 43,140 people are diagnosed (incidence 10–12: 100,000) with pancreatic ductal adenocarcinoma (PDAC) in the Unites States and the mortality rate of 36,800, almost equals this number [1]. PDAC ranks fourth on the list of cancer-related causes of death and despite extensive clinical and scientific effort, the prognosis of this exceptionally lethal disease has not improved significantly over the past decades. Surgical resection, for which only a minority (<20%) of patients qualify due to advanced stage of disease at time of diagnosis, is currently the only chance for cure, improving five-year survival rates from <4% if left untreated to 25–30% after resection [2–4]. Though of marginal impact, chemo(radiation) therapy administered in (neo)adjuvant setting has been shown to increase short-term survival rates in resectable and advanced stage disease [5–7]. Despite subtle progress over the years in terms of therapeutic strategies, no major new treatment options have come forward from numerous clinical trails. Nevertheless, much progress has been made in understanding the pathogenesis of PDAC during the past decades, suggesting that different therapeutic strategies based on these new insights are on the horizon [8–10]. 

PDAC, like all cancers, is fundamentally a genetic disease caused by alterations in cancer-associated genes. The identification of such specific mutated genes is critical for understanding the pathogenesis of PDAC. Nevertheless, one cannot achieve a reasonable overview by considering only individual genes in a cancer cell because the neoplastic potential of this cell is the end product of mutations in multiple genes and changes in multiple pathways that interact and reinforce each other. The rapidly expanding knowledge of genetic and molecular alterations and their role in pancreatic carcinogenesis has led to the question whether it is possible to design a patient-specific therapy based on the genetic fingerprint of an individual tumor. Since an increasing focus exists on designing these so-called targeted treatment strategies, this paper is aimed to put genetic alterations pancreatic cells undergo during malignant transformation in the context of their role in signaling pathways. In addition, this paper provides an update of the most recent advances made in the development of the targeted treatment approach in PDAC.

## 2. Precursors of PDAC

The development of invasive carcinoma in the pancreas is a stepwise process. Similar to colon cancer, noninvasive stages have been identified in PDAC preceding invasive carcinoma [11]. In recently published research, the clonal evolution of the earliest genetic alterations in tumor initiating cells towards frankly invasive and metastasized PDAC was followed and these studies indicated that such tumor progression takes at least more than a decade [12, 13]. This creates an important window of opportunity for early detection and much effort is put into attempts to map the genetic changes that take place in the pancreatic ductal cells of precursor lesions before they become invasive. 

Since 2004, there have been clear guidelines for classifying these precursor lesions of PDAC and three different types have been identified: pancreatic intraepithelial neoplasia (PanIN), mucinous cystic neoplasia (MCN), and intraductal pancreatic mucinous neoplasia (IPMN) [14]. MCN and IPMN are considered separate and specific entities that fall beyond the scope of this review [15, 16]. By far, the most common and also the generic precursor lesion of PDAC is the PanIN lesion. PanINs are found in the smaller pancreatic ducts and based on the degree of dysplasia reflected in the cytonuclear atypia and architectural change can be classified in four grades: PanIN-1A, PanIN-1B, PanIN-2, and PanIN-3. The least severe abnormalities are seen in PanIN-1 lesions; minimal cytonuclear atypia is present and cell polarity is retained with a basally located nucleus. The difference between PanIN-1A and -1B is that the cells in PanIN-1A lesions are flat, whereas the cells in PanIN-1B lesions are arranged in a micropapillary architecture. PanIN-2 lesions are characterized by evident cytonuclear atypia and infrequent mitoses. PanIN-3 lesions, also called carcinoma-*in-situ*, demonstrate all of the hallmarks of cancer including loss of polarity, nuclear atypia, frequent mitoses, and budding of groups of cells in the lumen. Yet, the lesion is confined within the basement membrane and no invasive growth is present [14]. The increasing grades of dysplasia in the various PanIN lesions manifest the morphological steps of tumor progression that precede invasive PDAC. These consecutive steps of tumor progression are genetically accompanied by a cumulative occurrence of specific and generalized molecular genetic alterations. Typically, an interplay between mutations in tumor-suppressor genes, oncogenes, and genome maintenance genes ultimately results in the development of PDAC. 

**Figure 1:**
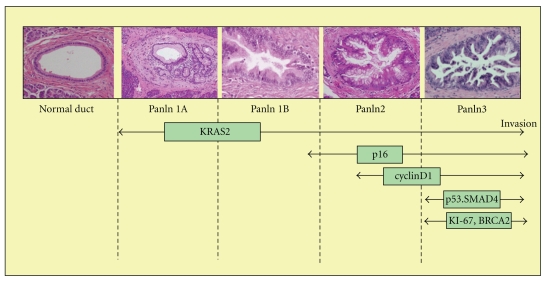
Progression model of pancreatic ductal adenocarcinoma from normal epithelium to invasively growing tumor. The progression is associated with the stepwise accumulation of specific genetic alterations depicted below the pictures.

Telomere shortening is considered the initiating event in pancreatic tumorigenesis by inducing genetic instability and is discussed separately below. Another early event in PDAC development is mutation of the oncogene *KRAS2,* which is found altered in 20% of PanIN-1 lesions and this percentage increases with progression to invasive carcinoma. The tumor suppressor gene most commonly found mutated in PDAC is *CDKN2A*. This gene is found mutated in, respectively, 30%, 50%, and 70% of PanIN-1, PanIN-2, and PanIN-3 lesions [17]. Two additional important tumor suppressor genes in PDAC are *TP53 *and* SMAD4*. In precursor lesions, mutations in these genes are mainly observed in PanIN-3 lesions in transition to invasive growth warranting *TP53/SMAD4* defects as a late event in PDAC development [18, 19]. In Figure 1 and Table 1, the most commonly observed specific genetic alterations in preinvasive lesions of PDAC are mentioned, but many additional alterations exist. In order to understand pancreatic carcinogenesis, the whole known spectrum of alterations has to be considered as well as the cellular interactions.

## 3. Molecular Characteristics and Regulatory Pathways in PDAC

**Figure 2:**
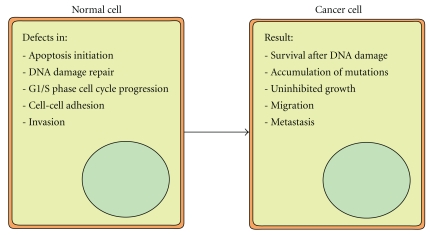
Cellular functions affected in pancreatic cancer.

In 2008, Jones et al. used global genomic sequencing to identify the genetic alterations in pancreatic cancer cells. Over 21,000 genes were screened in 24 different PDAC samples. On average, 63 relevant genetic alterations were found per sample, emphasizing the extreme complexity of this disease. These genetic alterations mostly affected 12, partially overlapping, signaling pathways that consequently contained abnormalities in the majority of cases [20, 21]. The identification of these pathways intelligently created a comprehensible view of pancreatic carcinogenesis without simplifying too much [21]. All previously known genetic alterations were included and put into the context of the pathways in which they function. Five of the pathways describe specific cellular functions; apoptosis, DNA damage repair, G1/S phase cell cycle progression, cell-cell adhesion and invasion (Figure 2). The other pathways are signaling cascades and can be divided into three groups: embryonic signaling pathways, the MAPkinase signaling pathways, and TGF-*β* signaling. The molecular characteristics of PDAC are described within the context of these various specific pathways in the subsequent paragraphs.  Table 2 gives an overview of the various affected pathways and their most commonly mutated genes in PDAC.

For the acquisition of an accumulation of genetic alterations by the neoplastic cells, genetic instability is a precondition [22]. Telomere shortening is considered as the initial neoplastic event that provides pancreatic epithelial cells the genetic instability that leads to the subsequently specific and generalized molecular alterations [23, 24]. 

### 3.1. Telomere Shortening

Telomere shortening is encountered in virtually all precursor lesions and invasive carcinomas [23, 25]. Telomeres are repeat sequences at the end of linear chromosomes that prevent fusion between the ends of these chromosomes. Pathologically short telomeres can result in ring and dicentric chromosomes that form so-called anaphase bridges during mitosis. Breakage of these anaphase bridges generates highly recombinogenic-free DNA ends, which in turn can result in chromosomal rearrangement. These cycles of chromosome bridging and breakage, called anaphase bridge-breakage-fusion cycles, repeat and thereby create the genetic instability that facilitates tumor development [26]. Telomerase, the gene that maintains telomere length, shows low expression during early pancreatic tumorigenesis before markedly increasing in the invasive tumor. The re-expression of telomerase probably restores genomic stability, enabling tumor progression by preventing further, possibly lethal, chromosomal damage [25, 27].

### 3.2. Apoptosis

**Figure 3:**
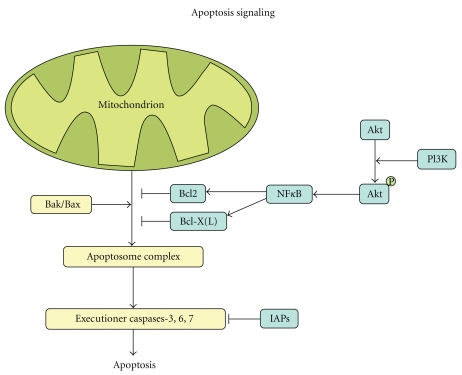
Apoptosis. Apoptosis is induced by executioner caspases upon activation by the apoptosome complex. This complex consists, among others, of cytochrome C, Caspase-9, and Apaf1 and is released from the mitochondria when proapoptotic signaling by Bak/Bax outweighs antiapoptotic signaling by Bcl2/Bcl-X(L). PI3K activates Akt through phosphorylation which subsequently activates NF-*κ*B. NF-*κ*B stimulates antiapoptotic signaling by Bcl2 and Bcl-X(L). Inhibitors of apoptosis proteins (IAPs) can inhibit apoptosis at the end of the signaling cascade by direct inhibition of the executioner caspases.

Apoptosis, or programmed cell death, plays an essential role in cancer development since resistance to apoptosis is a key factor in the survival of a cancer cell (Figure 3). Apoptosis is induced by executioner caspases upon activation by the apoptosome complex. This complex consists, among others, of cytochrome C, Caspase-9, and Apaf1 and is released from the mitochondria when pro-apoptotic signaling by Bak/Bax outweighs antiapoptotic signaling by Bcl2, Bcl-X(L) or Mcl-1. Inhibitors of apoptosis proteins (IAPs) can inhibit apoptosis at the end of the signaling cascade by direct inhibition of the executioner caspases. In PDAC, genes implicated in the apoptosis pathway were found altered in all tumors studied [21]. Also, previous reports document impaired apoptotic signaling in this disease. For example, a high fraction of apoptotic cells has been correlated with longer overall survival as well as absence of nodal involvement [28]. Moreover, most chemotherapeutics act through apoptosis induction whereby therapy resistance often is the result of defective apoptosis pathways. Antiapoptotic genes *BCL-2*, *BCL-X(L)*, and *MCL-1* are expressed in, respectively, 13%, 54%, and 86% of PDAC samples as shown by immunohistochemistry, and repression of *BCL-2* and *MCL-1* was shown to enhance apoptosis in PDAC [29, 30]. The observed apoptotic effect was even more pronounced when treatment was combined with gemcitabine [31]. 

NF-*κ*B is a transcription factor that regulates several different cellular mechanisms, of which most importantly apoptosis. NF-*κ*B stimulates antiapoptotic signaling by targeting *BCL-2 and BCL-X(L)* [32–34]. The NF-*κ*B signaling pathway is activated by a variety of different mechanisms in PDAC amongst others oncogenic K-ras signaling [35]. 

Oncogenic K-ras signaling also activates phosphatidylinositol 3-kinase (PI3K), another important protein in apoptosis. PI3K activates Akt through phosphorylation which subsequently activates NF-*κ*B. The *AKT2 *gene located on chromosome 19q is amplified in 10–20% of pancreatic cancers [36, 37], whereas PI3K/Akt signaling is activated in approximately 60% of PDACs [38]. PI3K signaling also involves mammalian target of rapamycin (mTOR), a downstream target of Akt. Activation of mTOR has been observed in approximately 75% of PDACs [39]. Therefore, inhibition of mTOR is an interesting target for therapy for which there are currently FDA approved inhibitors on the market. Although the exact role of the PI3K/Akt/mTOR pathway in pancreatic cancer remains to be elucidated, signaling of this pathway was shown to inhibit apoptosis, and inhibition of the pathway increased cellular sensitivity to gemcitabine [40, 41].

### 3.3. DNA Damage Repair

DNA damage control genes are responsible for safeguarding the integrity of DNA as they code for proteins that repair any damage that occurs in the cell during its lifespan. An important DNA damage repair gene is *TP53*, a tumor suppressor gene located on chromosome 17p that is frequently disrupted in many different human malignancies. *TP53* expression is lost in 50–75% of PDACs [42, 43]. P53 is involved in the cellular response to genotoxic stress where it mediates cell cycle arrest and apoptosis upon DNA damage. Therefore, loss of *TP53* signaling results in a decrease in apoptosis and increases the opportunity for genetic alterations to accumulate in the cells. 

Germline *BRCA2* gene mutations are responsible for ~10% of familial pancreatic cancer but mutations in this gene are also observed in approximately 7–10% of sporadic PDAC. The BRCA2 protein is involved in DNA damage repair, especially interstrand cross-linking repair [44–46]. The BRCA2 gene will be further discussed in the paragraph on hereditary PDAC.

A third group of DNA damage repair genes involved in the development of PDAC is the mismatch repair family (MMR) of genes. The MMR proteins target base substitution mismatches and insertion-deletion mismatches that arise as a result of errors occurring during replication. Alterations in the mismatch repair genes *MLH1, MSH2, MSH6,* and *PMS2 *eventually lead to microsatellite instability (MSI) and this genetic instability makes the genome vulnerable for the accumulation of other, more specific genetic alterations. Tumors of the pancreas with MSI are relatively rare compared to other malignancies of the digestive tract and are found in only 5% of pancreatic carcinomas. Pancreatic cancers with MSI have a distinct microscopic morphology that resembles their counterpart in the colon and are similarly called medullary type carcinomas [47–49]. Remarkably, microsatellite instable tumors have a significantly better prognosis compared to their microsatellite-stable counterparts [47, 48]. PDACs with MSI exhibit a higher antitumor reaction by T-lymphocytes and this could possibly be the reason for a better outcome [49].

### 3.4. G1/S Phase Cell-Cycle Progression

Cell-cycle progression and regulation is affected in virtually all cancers as is the case for PDAC. Alterations in genes regulating G1/S-phase transition play an important role in facilitating the uncontrolled growth rate of cancer cells. The most commonly affected tumor suppressor gene in PDAC involved in G1/S phase transition is the *CDKN2A* gene [50, 51]. This gene is located on the short arm of chromosome 9 (9p21) and is known for its involvement in hereditary melanoma when mutated in the germline [52]. The gene is inactivated in >90% of all PDACs, either by homozygous deletion (40%) or an intragenic mutation combined with loss of heterogeneity of the remaining wild type allele (40%) [50, 53]. Promoter hypermethylation is the cause for loss of *CDKN2A* function in 15% of the cases [50]. P16, the protein product of CDKN2A inhibits phosphorylation of Rb-1, thereby preventing G1/S transition and acting as an inhibitory cell-cycle regulator [54]. Loss of p16 expression therefore leads to uncontrolled G1/S transition and unregulated cell division, which facilitates tumor progression [55]. 

Other genes involved in cell cycle progression that occasionally show alterations in PDAC are *FBXW7, CHD1* and *APC2*, although much less frequently than *CDKN2A* [21, 56].

### 3.5. Cell Adhesion and Invasion

In normal pancreatic tissue, cells are anchored to each other and their surroundings via multiple connections. A decrease in these interactions can allow cells to detach from their surrounding and migrate/metastasize. As such, cell to cell adhesion and interaction play an important role in carcinogenesis. The connection between epithelial cells is mostly mediated by the adherent junctions composed of E-cadherin and catenins. E-cadherin proteins interlock with each other in the extracellular space, while intracellularly the E-cadherin protein is bound to actin filaments through catenins. Reduced expression of E-cadherin and *α*- and ß-catenins was demonstrated in approximately 60%, 40%, and 60% of pancreatic cancer samples, respectively [57, 58]. Reduced expression of E-cadherin is correlated with tumor dedifferentiation and correlates with tumor stage and lymph node involvement [57, 59]. Not only is the interaction between epithelial cells important in the preservation of the integrity of the tissue, the interactions between the neoplastic cells and extracellular matrix also play an important role, especially in PDAC, because stromal tissue surrounds most tumor cells. Integrins comprise a large family of cell surface receptors and they act as a bridge between the extracellular matrix (ECM) and the cytoskeleton [60]. These integrins direct cell migration and play an important role in invasion. In addition to this, integrins also regulate cell proliferation and apoptosis. Integrin-ECM interactions are vital for cell survival but since apoptosis pathways are often affected in PDAC, loss of the interaction does not necessarily lead to apoptosis in cancer cells. Integrin signaling can activate the ERK, JNK MAPK pathway and the PI3K pathway, important pathways in pancreatic tumorigenesis. In approximately two-thirds of the PDAC cases, a defect in integrin signaling can be identified [21]. Different components of integrin signaling can be deregulated; for example, Integrin *α*6*β*1 expression has been correlated with metastatic behavior in pancreatic cancer cell lines [61]. Furthermore, Niu et al. investigated the role of *α*v*β*6 integrin in PDAC and found that *α*v*β*6 inhibition resulted in a significant reduction in cell proliferation and invasion. Apoptosis was induced and more remarkably, *α*v*β*6 integrin knockdown increased gemcitabine sensitivity [62]. 

Another group of proteins involved in cell adhesion is the a-disintegrin and metalloproteinase (ADAM) protein family. ADAM proteins are cell surface proteins that activate MAPK pathways and integrin signaling through the release of growth factors. ADAM proteins have the ability to cleave ECM components and influence integrin/ECM interactions. ADAMs have only recently caught attention, thus not much is known about the specific role these proteins play in pancreatic carcinogenesis, though upregulation of different ADAM proteins has been reported in PDAC [63, 64]. Jones et al. found genetic alterations in various different ADAM proteins [21]. Because ADAM proteins influence many different substrates through autocrine and paracrine signaling, they may comprise promising new targets for therapy development.

### 3.6. MAPK Signaling Pathways 

**Figure 4:**
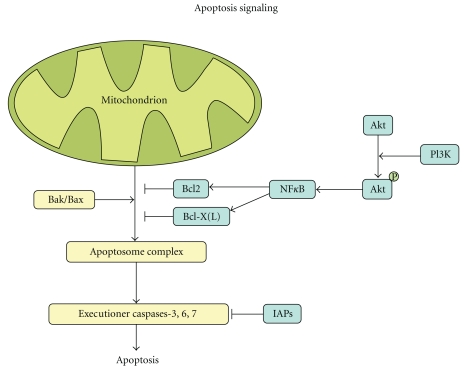
MAPkinase signalling. Mitogen-activated phosphorylated kinase signaling occurs through a common pathway. A cellular stimulus results in phosphorylation of MAP3K, which in turn phosphorylates MAP2K. MAP2K subsequently phosphorylates MAPK, resulting in altered transcription of the MAPK target genes. The different components of the two MAPK signaling pathways often affected in PDAC, the ERK pathway and the JNK pathway, are depicted in the boxes on each side of the signaling cascade.

There are three major mitogen activated phosphorylated kinases (MAPK): extracellular signal-regulated kinase (ERK), c-Jun N-terminal kinase (JNK), and p38. All MAPK signaling pathways consist of the same basic kinase components. Stimulation of an upstream MAP2K kinase (MAP3K) by growth factors, stress, or other extracellular signals leads to phosphorylation of a MAPK kinase (MAP2K), culminating in the phosphorylation of a terminal MAPK (Figure 4). 

The most influential of the three MAPK pathways in PDAC is the ERK pathway. It consists of the Raf protein (MAP3K) that phosphorylates MEK (MAP2K), which in turn phosphorylates ERK (MAPK), the latter influencing transcription of different target genes. This signaling cascade results in the activation of multiple oncogenic cellular functions. The most commonly mutated oncogene in PDAC is the *KRAS2* gene, of which the protein product Ras is an upstream activator of ERK signaling. *KRAS2* is located on chromosome 12p and the protein has an intrinsic GTP-ase activity. In PDAC, the gene is virtually always activated by a point mutation in codon 12, the GTP binding domain, leading to a constitutively active Ras protein [65, 66]. Therefore, it can be considered a molecular switch that in this fashion remains in the “on” position firing its oncogenic stimuli. As said before, *KRAS2* gene mutations are an early phenomenon in the development of PDAC, and the *KRAS2* gene is mutated in ~95% of PDACs. Interestingly, the few tumors that contain wild-type *KRAS2* often have a mutation in the *BRAF* gene, an oncogene located on chromosome 7q. The *BRAF* gene is mutated in approximately 5% of the PDACs and Raf, the protein product of *BRAF*, is a downstream target in the Ras signaling pathway. This explains the mutually exclusive nature of *KRAS2* and *BRAF *mutations in PDAC [56]. The high frequency and early nature of *KRAS2* mutations suggest an initiating role in PDAC development as confirmed in several studies on genetically engineered mice [67, 68]. Besides the effects Ras has on the ERK-pathway, Ras also influences multiple other genes among which NF-*κ*B and PI3K/Akt as discussed above.

The second MAPK pathway often affected in PDAC is the JNK pathway. In the whole genome sequence analysis study by Jones et al. mentioned earlier, in all but one of the sequenced samples a genetic alteration in the JNK pathway was identified [21]. In this signaling cascade the MAP3Ks, Ask1, MEKK1, and MLK phosphorylate the MAP2Ks, MKK4, and MKK7 which in turn phosphorylate JNK. The JNK pathway becomes activated upon cellular stress but more importantly, the pathway is activated by proinflammatory cytokines such as tumor necrosis factor *α* (TNF-*α*) or interleukin 1 (IL1) [69]. *MKK4* expression is lost in approximately 4–15% of PDACs [70, 71]. Remarkably, the JNK pathway has both tumor suppressor and oncogenic functions that have to be further investigated. Also it should be noted that the Kras and the JNK pathways interact; phosphorylation of JNK is partly responsible for induction of angiogenesis through Kras [72]. Recent studies have also connected MKK4 and its downstream targets (JNK and p38) to the TGF-*β* pathway.

### 3.7. TGF-*β* Pathway

**Figure 5:**
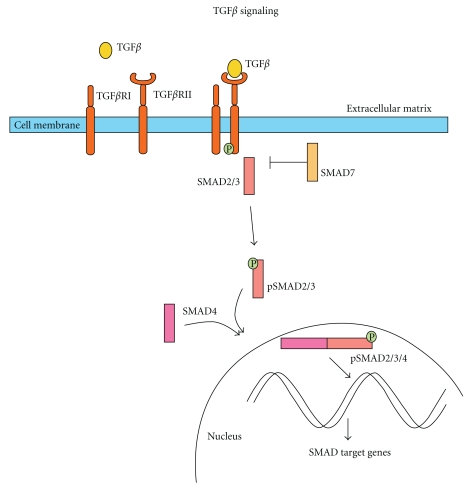
The TGF-*β* signaling pathway. The TGF-*β* signaling pathway is activated by binding of TGF-*β* to a type II receptor, which facilitates the recruitment and phosphorylation of the type I receptor. This pTGF-*β*RI activates either SMAD2 or SMAD3 by phosphorylation. The phosphorylated SMAD2/3 forms a complex with SMAD4 and transports to the nucleus where it influences SMAD target gene transcription. SMAD7 can inhibit TGF-*β* signaling through inhibition of TGF-*β*RI phosphorylation.

The transforming growth factor *β* (TGF-*β*) pathway has been linked to PDAC for many years. TGF-*β* signaling is involved in a wide range of cellular processes [73]: It is one of the most potent cell proliferation inhibitors and has many other cellular responsibilities including differentiation, apoptosis, and angiogenesis [74]. Binding of a TGF-*β* family ligand to the TGF-*β*II receptor leads to phosphorylation of the TGF-*β*I receptor and thereby activation of the TGF-*β* receptor substrates capable of signal transduction, that is, the Smad family proteins. Eight different *SMAD* genes have been described. Once activated, the receptor subsequently phosphorylates a regulatory Smad (Smad1–3, 5, 8), allowing this protein to associate with Smad4. The latter aids the regulatory Smad complex in its transfer to the nucleus where subsequent transcription of the target genes is induced. Inhibitory Smads regulate Smad-signaling through inhibition of the TGF-*β* receptor phosphorylation. Thus far, Smad7 is the only characterized inhibitory Smad (Figure 5) [73].

**Figure 6:**
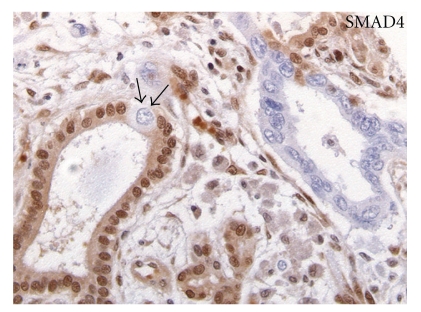
SMAD4 immunohistochemistry. Loss of *SMAD4* expression is clearly depicted in the PDAC cells. Arrows: single cell with clear histological changes exhibiting SMAD4 loss, surrounded by *SMAD4* wild-type cells.

TGF-*β* influences cellular proliferation through inhibition of G1/S-transition. This is accomplished though expression of cyclin kinase inhibitors such as p15, p21, and p27 [75]. Also, TGF-*β* signaling represses c-Myc expression, an ubiquitous promoter of cell cycle progression. Jones et al. found altered TGF-*β* pathway expression in all their PDAC samples [21]. The most commonly affected protein in the TGF-*β* pathway is Smad4. Smad4 is inactivated in ~55% of PDACs [76, 77]. Patients with preserved Smad4 signaling have a significantly longer survival than patients with Smad4 loss [77–79]. Also, Iacobuzio-Donahue et al. found a significantly higher percentage of Smad4 loss in patients who had died from PDAC with widespread metastatic disease compared to patients who died of locally advanced tumors [79]. Loss of Smad4 expression is not only a prognosticator but it can also serve as a diagnostic biomarker since sensitive and specific antibodies are available that can be used to characterize Smad4 protein expression by immunohistochemistry (Figure 6) [78]. 

Other proteins in the TGF-*β* pathway that are occasionally found altered in PDAC are the TGF-*β*RII (4%) and TGF-*β*RI (1%) [80]. Apart from binding to the TGF-*β*R, TGF-*β* ligands can also activate other signaling pathways including the MAPK pathways ERK and JNK [81–83]. This depicts the fact that although TGF-*β* signaling has a tumor suppressive function in the normal epithelium, it can promote tumor progression in late disease stages. Further research has to be conducted to determine the true potential of this pathway for the development of targeting agents.

### 3.8. Embryonic Pathways

**Figure 7:**
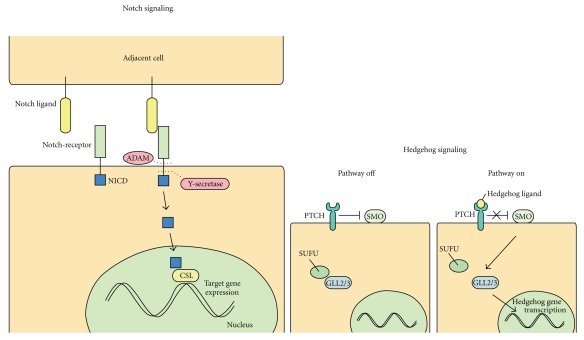
Left Notch signaling. Upon Notch ligand binding, ADAM performs the first cleavage quickly followed by the second cleavage performed by *γ*-secretase. This second cleavage releases the Notch intracellular domain (NICD) into the cellular lumen. NICD transports to the nucleus where it interacts with a transcription domain resulting in transcription of the Notch target genes. Right Hedgehog signaling. In normal pancreatic cells, Hedgehog signaling is repressed; the patched (PTCH) receptor represses the smoothened (SMO) receptor. Intracellularly, the hedgehog inhibitor suppressor of fused (SUFU) binds the GLI family zink finger transcription factors GLI2/3 thereby inducing proteasomal cleavage resulting in repressor forms of GLI2/3. When one of the Hedgehog ligands attaches to Patched, this abrogates the inhibition on Smoothened. Smoothened inhibits proteasomal cleavage of GLI2/3 and thereby facilitates transcriptional activity.

Not surprisingly, since embryogenesis shares many characteristics with carcinogenesis, different embryonic pathways are involved in tumor development. There are three embryonic pathways involved in pancreatic carcinogenesis: Notch, Hedgehog, and Wnt (Figure 7).

The Notch pathway plays an important role in pancreatic organogenesis, but after formation of the pancreas, signaling is largely restricted to a putative progenitor population known as centroacinar cells [84–87]. Several studies have shown upregulation of Notch pathway activity in PDAC and inhibition of this pathway resulted in decreased tumor proliferation and increased apoptosis [87–90]. Somatic point mutations in one of the four Notch-receptor genes do not seem to be the driving force behind altered Notch signaling in pancreatic cancer [90]. Still, 100% of the PDAC samples in a genome-wide sequencing study revealed alterations in the Notch pathway [21]. Notch signaling interacts with many other oncogenic pathways including the Hedgehog pathway, KRAS signaling and the NF-*κ*B pathway.

The second embryonic pathway often affected in PDAC is the Hedgehog (Hh) pathway. This signaling cascade plays an important role in the organogenesis of the gastro-intestinal tract. Surprisingly, Hh signaling is absent in the developing pancreas [91, 92] but the pathway is activated in 70% of PDACs [93]. Some of the Hh signaling targets are components of other signaling pathways involved in PDAC such as Wnt proteins, TGF-*β*, and CyclinD [94–96]. Although it has long been common knowledge that Hh signaling is active in PDAC, its exact role in tumorigenesis is unclear. It seems that neoplastic epithelial cells do not have the ability to react to Hh signaling. Instead, Hh ligands are expressed in the epithelial cells and it has been suggested that these affect the stromal compartment of a tumor through paracrine signaling. In one particular study, the strong desmoplastic reaction characteristic for PDAC was shown to be further enhanced when Hh signaling was activated [96]. In addition, inhibition of the Hh pathway decreased the total volume of orthotopically implanted tumors by inhibiting the stromal component in mice [97]. Another study showed that treatment with an Hh pathway inhibitor produced a clear decrease in tumor growth primarily through a decrease in number of stromal cells [98]. Similarly, disruption of Hh signaling in a transgenic mouse model increased response to chemotherapy [99]. This improved response was due to a diminished desmoplastic reaction and better accessibility of the tumor cells for the chemotherapeutic agent. In short, there seems to be an important role for Hh signaling in the stromal component of PDAC. Moreover, since the desmoplastic reaction has been related to resistance to therapy it warrants further investigation of Hh and its role in the development of PDAC. Clinical trials with inhibitors of hedgehog signaling are in progress.

The third embryonic pathway, Wnt, shows increased activity in approximately 30–65% of PDACs, and an increase in Wnt-target expression correlates with poorer differentiation and poor prognosis [100–102]. Active Wnt expression results in the transcription of different target genes including *CyclinD*, matrix metalloproteinase 7 (*MMP7*), and *c-MYC*. CyclinD is overexpressed in 65% of PDACs and stimulates G1/S transition. Expression of this protein is associated with poor prognosis [103]. MMP7, a member of the matrix metalloproteinase family, degrades extracellular matrix proteins, and MMP7 expression is implicated in metastases. Overexpression of this protein is found in practically all PDACs [104]. C-Myc is a transcription factor that regulates thousands of genes involving a large spectrum of cellular functions including cell proliferation, differentiation, death, and tissue reorganization. Amplification of *CMYC* is identified in 20–50% of all PDACs [105]. The Wnt signaling cascade can be activated through interactions with the Hh, NF-*κ*B, TGF-*β*, and Notch pathways [106–108].

## 4. Genetic Susceptibility

Approximately 5–10% of patients with pancreatic cancer have a positive family history for the disease. Having a first-degree relative with PDAC doubles the chance of developing pancreatic cancer compared to individuals without such a history, and the risk increases with increasing number of affected relatives suggesting a hereditary component. Some of these PDACs arise in the setting of a known familial cancer syndrome; however, in most instances the genetic basis for the familial aggregation is not known [109]. 

To date, at least 5 hereditary disorders that significantly increase the chance of pancreatic cancer development have been described. These include familial atypical multiple melanoma and mole (FAMMM) syndrome, Peutz-Jeghers syndrome, hereditary pancreatitis, familial breast cancer, and other syndromes related to alterations in Fanconi anemia genes, and the Lynch syndrome (Table 3). 

The FAMMM syndrome is caused by germline mutations in the *CDKN2A* gene. As stated before, this gene is often somatically mutated in sporadic pancreatic cancer. Patients suffering from this syndrome have a 20–34-fold risk of developing PDAC [110]. This risk is especially high when the mutation is a specific 19-base-pair deletion in *CDKN2A*: the p16-Leiden deletion [111]. 

Peutz-Jeghers syndrome is caused by mutations in the *STK11/LKB1 *gene, a serine threonine kinase involved in a large number of cellular functions, from control of cell polarity to metabolism. Patients suffering from this syndrome have a 132-fold increased risk of developing PDAC with a 30–60% lifetime risk of PDAC at age 70 [112–114]. 

Patients with hereditary pancreatitis develop recurrent episodes of pancreatitis, starting at a young age. The syndrome is most commonly caused by mutations in the cationic trypsinogen gene *PRSS1 * [115]. Another gene that is occasionally found altered in patients with hereditary pancreatitis is the serine peptidase inhibitor *SPINK1* [116]. Carriers of either of these mutations have a highly increased risk of developing pancreatic cancer with a lifetime risk of 25–40% at age 60. 

The two *BRCA* genes are best known for their role in familial breast and ovarian cancers but *BRCA2* also plays a role in pancreatic cancer development. Carriers of germline *BRCA2* gene mutations have a 3–10-fold increased risk of developing PDAC. A specific interest goes to the Ashkenazi Jewish population as approximately 1% of Ashkenazi Jews are carriers of a founder *BRCA2* mutation, 6174delT [117]. 

Fanconi anemia is a hereditary cancer susceptibility disorder, with occurrence of multiple haematological malignancies. The Brca2 protein interacts with different Fanconi anemia pathway components, and the corresponding encoding genes, especially *FANCC* and *FANCG, *have also been reported to increase the chance of PDAC development when mutated. Recently, *PALB2*, yet another FANC gene,was reported to be responsible for ~3% of the cases of familial pancreatic cancer [118, 119]. *PALB2* encodes a protein that enables the localization and binding of Brca2 to sites of double-strand DNA breaks.

Lynch syndrome is caused by germline mutations in a number of DNA mismatch repair genes. Patients suffering from the syndrome have a slightly increased chance of developing pancreatic cancer although there is still some debate about the exact role in PDAC development [120].

Identification of germline mutations in the previously discussed genes is of great importance, not only for screening purposes but also because they could potentially hold therapeutic consequences. Furthermore, no genetic basis for cancer susceptibility is identified in most cases of families exhibiting high numbers of PDAC affected individuals. More research on genetic susceptibility for PDAC will have to be conducted to explain the genetic basis for disease development.

## 5. Treatment of PDAC

Adjuvant therapy after resection of the tumor consisting of gemcitabine has been the treatment of choice since 1997 when it was shown to improve both disease-free and overall survival [5, 6]. Several studies examining the effect of adding other therapeutic agents to gemcitabine have been conducted over the past years with disappointing results [121, 122]. Only the addition of erlotinib showed slight improvement of overall survival [123]. A recently published report confirmed the earlier observed limited beneficial effect of adding erlotinib; however, the authors concluded that this was no justification for a phase III trail [124]. Reports comparing single-agent gemcitabine to adjuvant chemoradiation therapy have been inconclusive. Chemoradiation therapy has been implicated in the USA since the Gastro-Intestinal Tumor Study Group trial was published which showed longer overall survival in patients treated with adjuvant chemoradiation [125, 126]. In Europe, however, a similar study failed to find a significant survival advantage for the group receiving additional radiotherapy thus chemoradiation therapy did not become the standard treatment [127]. Although the most recent reports on this subject suggest a significant advantage for the addition of radiotherapy, there is still controversy about this subject and more research needs to be done before radiation therapy can be included as standard first-line of treatment for PDAC in Europe [125]. It has been suggested that neoadjuvant treatment with chemotherapy, radiation therapy, or chemoradiation therapy, could downstage borderline resectable tumors. Several recent studies have shown promising results for treating borderline resectable tumors with chemoradiation, enabling resection and approaching similar survival rates as truly resectable tumors [128–131]. This is still under investigation and future studies will have to be conducted to justify the use of neoadjuvant treatment. 

Approximately 80% of patients present with locally or systemically advanced disease-making resection redundant. For these patients, only palliative treatment options remain. Single-agent gemcitabine is currently recommended as standard first-line chemotherapy for patients with advanced disease [5]. 

Since the arrival of whole genome sequencing, it has become possible to identify all the genomic alterations that lead to the development of pancreatic cancer. The next logical step is to translate this knowledge into better treatment options. Until recently, no targeted agents were found to improve outcome in the clinical setting although many studies have shown promise in the *in vitro* setting. In the past year, a group used mutation analysis to guide their treatment strategy for the first time [132]. Earlier studies had shown that PDAC cell lines harboring mutations in the above-mentioned *BRCA2 *gene, but also other genes related to the Fanconi Anemia syndrome (*FANCC*, *FANCG*) responded better to treatment with interstrand cross linking (ICL) agents than *FANC/BRCA* wild-type tumors [133]. The *FANC/BRCA* pathway is involved in the repair of double stranded DNA-breaks. As ICL-forming agents induce this type of DNA damage, susceptibility of *FANC/BRCA* mutated tumors to the ICL-forming agents seemed reasonable. Showalter et al. performed mutation analysis for *BRCA2 *and one patient harboring a mutation in this gene was treated with cisplatin, an ICL agent, in addition to gemcitabine showing favorable results (the patient is still alive after 32 months). In theory, PDACs carrying *PALB2* mutations should be sensitive to the same targeted therapeutic as *PALB2* is a binding partner of *BRCA2*. Trials justifying use of ICL agents in *PALB2* mutated PDAC still have to be conducted.

We have recently seen a Peutz-Jeghers syndrome patient with pancreatic cancer whose tumor showed complete loss of *LKB1*, an inhibitor of mTOR. This patient responded to treatment with everolimus, one of the known mTOR inhibitors used in clinical setting. Specifically, the tumor diminished in size by more than 50% within 6 months but became resistant thereafter [134]. 

Inhibition of Kras signaling with farnesyl transferase inhibitors used in the past did not have a beneficial effect (reviewed by [135]). In 2010, a new therapeutic agent was identified targeting the Kras pathway. Protein Kinase C iota (PKC iota) was shown to drive transformed growth in pancreatic cancer cell lines via inhibition of oncogenic Kras activity, and inhibition of PKC iota resulted in a significant reduction of metastases and invasion in preclinical models [136]. Further research has to be done to map the effectiveness of inhibiting PKC iota *in vivo*. 

*MTAP*, a gene located near *CDKN2A*, is codeleted with the *CDKN2A* gene in 30% of the pancreatic cancers. *MTAP* might be a possible therapeutic target as approaches to selectively target cells with *MTAP* defects have already been developed [137, 138]. However, these have not been tested in a clinical setting yet. 

It seems logical that over the next few years multiple small steps, hopefully adding up to significant progress, will be taken on the road to targeted treatment of PDAC.

## 6. Conclusion

The aim of this review was to emphasize the complexity of tumorigenesis in pancreatic ductal adenocarcinoma and to provide an introductory overview of the pathways affected in PDAC. As the knowledge on tumorigenesis of PDAC expands rapidly, so do the possibilities to design more effective treatment. The arrival of genome sequencing has offered the opportunity to establish an overview of the genetic alterations that lead to tumor development and this could subsequently play an important role in our search for new therapeutic targets. The complexity of the genetics accompanying PDAC indicates that it is impossible to design a treatment that fits all. From this can be deducted that personalized treatment based on tumor genotyping will probably be most effective and feasible. The use of ICL agents for tumors harboring *BRCA2* mutations is the first step in that direction. 

Based on the data presented in this review, it seems advisable to shift the focus of research from most commonly affected genes to the most commonly affected pathways as some important yet rare alterations could be missed. The interactions all these pathways undergo are extensive and complex as mentioned earlier. When considering personalizing treatment, designing workable and quick tumor characterizing assays and targeting pathways rather than individual genes seems to hold the future of cancer therapy in PDAC.

## Figures and Tables

**Table 1:** Most commonly affected genes in PDAC.

Type	Gene	Cellular function	Affected in PDAC
Tumor suppressor genes	*CDKN2A/p16*	G1-S phase cell cycle inhibition	95%
	*SMAD4*	TGF*β*	55%
	*TP53*	Cell-cycle arrest	75%

Oncogenes	*KRAS2*	ERK-MAPkinase signaling	>90%
	*CyclinD*	Cell cycle progression	65%
	*BRAF*	ERK-MAPkinase signaling	5%

Genome maintenance genes	*MLH1/MSH2*	DNA damage (mismatch) repair	4%
	*BRCA2*	DNA damage repair	7–10%

**Table 2:** The 12 commonly affected signaling pathways in PDAC accompanied by the most commonly affected genes from these pathways.

Regulatory pathway	Affected genes
Apoptosis	*TP53*
DNA damage repair	*TP53*
G1/S transition	*CDKN2A/p16, CyclinD*
Cell-cell adhesion	
Regulation of invasion	
* Integrin signaling *	
*Homophilic cell adhesion *	*CDH1*
Embryonic signaling	
*Notch pathway *	
*Hedgehog pathway *	
*Wnt pathway *	
MAPK signaling	
* c-Jun N-terminal kinase *	
*ERK *	*KRAS2*
*TGF-*β* signaling *	*SMAD4*

**Table 3:** Hereditary syndromes associated with an increased risk of PDAC development.

Syndrome	Affected gene(s)	Relative risk of PDAC
Familial atypical multiple melanoma and mole syndrome	*CDKN2A*	20–34
Peutz-Jeghers syndrome	*LKB1*	>100
Hereditary pancreatitis	*PRSS1/SPINK1*	~90
Familial breast cancer	*BRCA1/2*	3–10
Lynch syndrome	*mismatch repair genes*	unknown

## Abbreviations

PDAC: Pancreatic ductal adenocarcinoma
PanIN: Pancreatic intraepithelial neoplasia
MCN: Mucinous cystic neoplasia
IPMN: Intraductal pancreatic mucinous neoplasia
PI3K: Phosphatidylinositol 3-kinase
mTOR: Mammalian target of rapamycin
MMR: Mismatch repair
MSI: Microsatellite instability
ECM: Extracellular matrix
ADAM: A-disintegrin and metalloproteinase
ERK: Extracellular signal-regulated kinase
JNK: C-Jun N-terminal kinase
MAPK: Mitogen activated phosphorylated kinases
Hh: Hedgehog
TGF-*β*: Transforming growth factor *β*
TNF-*α*: Tumor necrosis factor *α*
IL1: Interleukin 1
MMP7: Matrix metalloproteinase 7
FAMMM: Familial atypical multiple melanoma and mole
ICL: Interstrand crosslink
PKC iota: Protein kinase c iota.
